# The impact of platelet function or C-reactive protein, on cardiovascular events after an acute myocardial infarction

**DOI:** 10.1186/1477-9560-7-12

**Published:** 2009-07-07

**Authors:** Angelo Modica, Fredrik Karlsson, Thomas Mooe

**Affiliations:** 1 Department of Internal Medicine, Section of Cardiology, Östersund Hospital, Östersund, Sweden; 2 Department of Public Health and Clinical Medicine, Umeå University Hospital, Umeå, Sweden

## Abstract

**Background:**

Recurrent cardiovascular events following acute myocardial infarction (AMI) are common. The purpose of this study was to evaluate the impact of platelet aggregation, PFA-100 closure times and peak C-reactive protein (CRP), respectively, on the occurrence of death, myocardial infarction and ischemic cerebral events after an AMI. Furthermore, to examine the relationship between the platelet function tests and peak CRP.

**Methods:**

Three hundred and thirty-four patients with AMI were included in the study. Platelet aggregation was analyzed by an aggregometer using laser light (PA-200). The state of high residual platelet reactivity was defined as normal closure times (PFA-100) during treatment with aspirin.

**Results:**

The fourth quartile of peak CRP was associated with poorer outcome as compared to the first quartile in a multivariate Cox-regression analysis, with a hazard ratio of 2.0 (95% CI 1.1–3.7) for the occurrence of death, myocardial infarction and ischemic cerebral events. The fourth quartile of peak CRP (>64.6 mg/l) was associated with platelet aggregation (p < 0.001, adjusted R^2 ^= 0.13) and high residual platelet reactivity, in a multivariate model, with an odds ratio of 2.9 (CI 95% 1.3–6.8), as compared to the first quartile. Neither the highest quartile of platelet aggregation nor the state of high residual platelet reactivity predicted new cardiovascular events.

**Conclusion:**

In patients with myocardial infarction, measured peak CRP is associated with new cardiovascular events. Despite an association with peak CRP neither more pronounced platelet aggregation nor PFA-100 closure times independently predict new cardiovascular events.

## Introduction

Recurrent cardiovascular events following acute myocardial infarction are common despite the availability of modern anti-thrombotic and anti-platelet treatment [1-5]. Although outcomes such as heart failure and recurrent AMI seem to be declining over time, as treatments for myocardial infarction improve [6-9], the issue of insufficiently inhibited platelets persists. In addition, the fact that stroke occurrence is high early after an AMI and declines rapidly with time suggests that the systemic inflammatory reaction plays a role [2,4].

The tissue damage in myocardial necrosis leads to an inflammatory reaction which is reflected for example, in increased levels of C-reactive protein (CRP), cytokines and fibrinogen [10,11]. The peak value of CRP during the hospital stay is related to the extent of the inflammatory reaction [12], which may be associated with platelet activation. In patients with AMI, CRP is positively correlated with expression of COX-2 in monocytes [13], which could hypothetically promote platelet activation via Thromboxane A2. Elevated CRP on admission is associated with poorer outcome in the acute coronary syndrome and among healthy individuals [14-17], but the relation of peak CRP concentration, during the course of AMI, to platelet reactivity and the risk of subsequent cardiovascular events has never been explored.

The primary purpose of this study was to evaluate the impact of measured peak platelet aggregation, high residual platelet reactivity, defined from PFA-100 closure times, and measured peak CRP, respectively, on the occurrence of cardiovascular events after an acute myocardial infarction. Furthermore, the relationship between the platelet function test values and measured peak CRP was examined as a secondary purpose.

## Materials and methods

### Study population

Three hundred and thirty-four patients with AMI, admitted to the cardiac intensive care unit at Östersund Hospital during the period January 1, 2002 to October 30, 2003, were included in the study. Östersund Hospital is the primary hospital for the County of Jämtland with a catchment area of approximately 128,000 inhabitants. All patients with a diagnosis of AMI during the inclusion period were considered for inclusion. An in-hospital stay of at least three days, aspirin treatment on discharge and complete PFA-100 and peak CRP data were required for inclusion. Another 163 patients with AMI were admitted to the hospital during the inclusion period but were not included because of death (*n *= 19), discharge before the required three in-hospital days (n = 71), not being on aspirin treatment on discharge (29) or incomplete laboratory data (n = 64). There could be more than one reason for a particular patient to be excluded. An acute myocardial infarction was diagnosed according to the guidelines of the European Society of Cardiology [18]. Patients receiving any treatment for diabetes were classified as diabetics. No glucose tolerance tests were performed. Patients were classified as hypertensive if they had a diagnosis of hypertension and as smokers if they were current smokers. Any histories of atrial fibrillation or atrial fibrillation during current hospitalization were classified as atrial fibrillation. Any past history of ischemic cerebral event (not necessary verified by x-ray) was classified as previous stroke. An ischemic cerebral event was defined as the occurrence of an ischemic stroke or a transitory ischemic attack (TIA). Heart failure during the hospital stay was defined as the need for intravenous furosemide or a bedside diagnosis of heart failure (Killip class > 1). Conventional anti-platelet and anti-thrombotic treatments were used as clinically indicated. The routinely used aspirin dose was 75 mg per day. This study complies with the Declaration of Helsinki and was approved by the local ethics committee. Informed consent was obtained from all subjects.

### Primary endpoint and secondary aim

The primary endpoint of the study was the composite of ischemic cerebral event, new myocardial infarction and death from all causes. The median follow-up duration was 44 (interquartile range 35–55) months. Survival confirmation and date of deaths were obtained from the Swedish National Population Registry. All survivors were contacted for a telephone interview concerning possible cardiovascular events. Ischemic cerebral events and myocardial infarctions were verified from hospital records. As a secondary aim we analyzed the level of measured peak CRP concentration in relation to measured peak platelet aggregation and high residual platelet reactivity.

### Blood tests

Blood samples were obtained via venipuncture and collected in tubes containing sodium citrate (0.129 M) on admission and on days two, three and five in hospital for the PA-200 analysis and on the third day for the PFA-100 analysis. A blood sample collected in serum tubes for CRP analysis was obtained on days one, two, three and five after admission. The measured peak values of PA-200 and CRP were used in this study. CRP was measured with C-reactive protein (latex) high sensitive immunoturbidimetric assay on Modular Analytics System (Roche). The coefficient of variation for the CRP analysis was 4.0% and 1.3% at the CRP-concentration level 0.94 mg/L and 17.1 mg/l respectively. The peak value of consecutive troponin T analyses was recorded. A blood sample for troponin T analysis was taken routinely every 8 hour. Troponin T was measured by an electrochemiluminiscence immunoassay (e170 Modular). GFR calculations were based on measurements of cystatin C levels for each patient. Plasma cystatin C measurements were performed by means of a particle enhanced immunoturbidimetric method (Modular P). LDL was calculated from the serum concentrations of cholesterol and fasting triglycerides using the Friedewalds formula. Von Willebrand factor was measured with an ELISA using reagents from DAKO (Copenhagen, Denmark). The test results (PA-200, PFA-100) were not accessible by the attending physicians.

### PFA-100 system testing

The PFA-100 (Platelet Function Analyzer, Dade Behring, Leiderbach, Germany) measures platelet function as primary hemostasis capacity in citrated whole blood [19]. The cartridge with collagen and epinephrine (CEPI) was used in the present study. The function of PFA-100 has been well documented and is not further presented here [20]. We defined the state of high residual platelet reactivity as a normal closure time (CT) value even when the subject was taking aspirin [21].

### Control group and reference values

For interpretation of the PFA-100 results, in house reference ranges were established from analyses in a control group of 278 volunteers without known cardiovascular disease (mean age 58.5 years, range 30 to 93). These individuals had no previous history or laboratory results indicative of platelet dysfunction, meaning no anaemia, no bleeding diathesis and normal platelet count. The use of non-steroidal anti-inflammatory drugs was not allowed. Since it is not unusual to find persons with mild von Willebrand disease in a selected volunteer population, and because individuals might be unaware that they have actually taken aspirin-like substances, we discarded all CT-values above the 95^th ^percentile before defining the normal range. The reference range (90–197 sec) was then determined based on a 90% central interval of the results. The coefficient of variation was calculated at 15.3% for the collagen and epinephrine cartridge (CEPI).

### PA-200 system testing

The PA-200 aggregometer (Kowa Inc, Tokyo, Japan) has been described in detail by Ozaki and co-workers [22]. It is a particle counting technique based on laser light scattering (LS). The validity of this method has been confirmed in several studies [23-27]. A 20-mW diode laser generates a laser beam measuring 40 μm (wave length 675 nm) in diameter, which is passed through 300 μl of platelet-rich plasma (PRP) stirred in a cylindrical glass cuvette with a 5 mm internal diameter. Light scatter generated by single platelets and platelet aggregates is measured within a region of approximately 30 × 65 × 145 μm by a photocell array. The signal frequency is recorded at 10-second intervals. The light scattering intensity increases in proportion to the particle size in a suspension and thus provides an estimate of platelet aggregate size in PRP. Data are expressed as the change over time (s) in the number of aggregates (counts/10 sec) of individual sizes (determined by light intensity, expressed in volts). The LS signals are digitized with an A/D converter and processed by a computer. Data are recorded as a two-dimensional graph showing the change over time (s) of the cumulative number of aggregates (counts/10 s during 10 min). Particles with an intensity of 25 to 400 mV represent small aggregates (9–25 μm), which are used to study platelet activation. To obtain platelet-rich plasma, the blood samples are centrifuged at 150 g for 10 min at room temperature. The remaining blood samples are then centrifuged at 300 g for 10 min to obtain platelet-poor plasma, which is used as a reference. As an agonist, we used 30 μL of a solution containing 0.1 mg epinephrine/L [24].

### Calculation and statistics

Data were analyzed using SPSS 13.0 software. Group data are expressed as rates for variables on a nominal scale. Differences between two groups were assessed with the Mann-Whitney U test. Differences between multiple groups were assessed with the Kruskal-Wallis test. Differences between proportions were analyzed with the chi-square test. Troponin T and CRP values were divided into quartiles in the analyses. Repeated measurements of platelet activation during hospitalization were analyzed with the non-parametric Friedman test. Independent predictors of platelet aggregation measured by the PA-200 were identified using a multiple linear regression model. Age, CRP, troponin T, the use of low molecular weight heparin (LMWH), and the use of clopidogrel were considered to be of potential importance and included in the models. An approximate normal distribution with improved skewness and kurtosis was achieved using the square root transformation of the PA-200 results. Independent predictors of high residual platelet reactivity were identified using a multivariate logistic regression model. The variables of age, CRP, troponin T, platelet count, von Willebrand factor, treatment with thrombolysis, the use of LMWH and the use of clopidogrel were included in the model. A power analysis indicated that in a model with a binary independent variable an OR = 2 could be identified with 80% power with a sample size of n = 250, p = 0.05. One hundred and sixty-seven were classified as high residual platelet reactivity, providing sufficient precision for the number of covariables in the model (at least 10 events per independent variable). The relationship between peak CRP and closure times was investigated using Spearman's rank order correlation. We used Cox-regression analysis to calculate the unadjusted and adjusted relative hazard ratios and 95 percent confidence intervals for stroke, myocardial infarction and death of any cause in relation to peak CRP, platelet aggregation and the state of high residual platelet reactivity. The co-variables age, sex, smoking status, diabetes, atrial fibrillation, clinical heart failure (Killip class > 1), baseline glomerular filtration rate (GFR), troponin T, platelet aggregation, high residual platelet reactivity and intervention with CABG (CoronAry By-pass Grafting) or PCI (Percutanous Coronary Intervention) were included in the adjusted model. One hundred and forty-one events occurred, providing sufficient precision for the number of covariables in the model (at least 10 events per independent variable). Neither use of clopidogrel and low molecular weight heparin during hospitalization nor medications at discharge had any discernible effects in the multivariate models. The assumptions for Cox-regression analysis were evaluated by Kaplan-Meier curves for all the variables included. The null hypothesis was rejected for values of *p *< 0.05.

## Results

The mean age of the study population was relatively high (72 years). Almost one third of the subjects had a history of previous myocardial infarction. Sixteen percent had a history of ischemic stroke. Twelve percent had a diagnosis of heart failure and 20 percent had diabetes mellitus prior to admission. Twenty-one percent were current smokers. The median time from the onset of the episode of chest pain to blood sampling was seven hours.

There were important differences in the clinical characteristics between the different quartiles of peak CRP (Table 1). Patients with higher peak CRP levels were older, had more impaired kidney function and more often had a history of heart failure. They were also less likely to receive an intervention (PCI or CABG) after the AMI. Medical treatment with clopidogrel, ACE-inhibitors and statins also differed between quartiles, which reflect differences in age of the patients and type of myocardial infarction. Among those with higher peak CRP levels there was a higher rate of thrombolysis. Clopidogrel was not part of the therapeutic regime in ST-elevation myocardial infarction. Clinical heart failure and treatment with ACE-inhibitors were more common among those with highest peak CRP concentration, probably reflecting more extensive myocardial infarction. There were no differences between the groups in the time between symptom onset and first blood sampling.

**Table 1:** Clinical characteristics and medical treatment according to quartiles of peak C-reactive protein

	**Quartile of C-reactive protein, (Range mg/l)**				
					
	1^st ^(n = 85) (0.05–5.3)	2^nd ^(n = 82) (5.4–19.6)	3^rd ^(n = 84) (19.7–64.5)	4^th ^(n = 83) (64.6–304)	p
Female, %	35	42	27	29	0.2

Age, mean (range)	70 (41–91)	70 (27–90)	73 (43–97)	75 (53–92)	0.02

Waist circumference, cm (SD)	95 (12)	96 (13)	96 (12)	95 (11)	1.0

Current smokers, %	19	18	25	22	0.7

Hypertension, %	29	44	35	45	0.1

Diabetes, %	16	24	26	12	0.07

Previous AMI, %	25	30	28	25	0.8

Previous stroke, %	11	15	18	13	0.6

Previous heart failure, %	11	15	8	18	0.2

Atrial fibrillation	16	16	23	23	0.5

GFR, mean (SD)	85 (33)	84 (36)	81 (35)	66 (35)	0.004

TnT peak, median μg/L	0.49 (0.21–1.26)	1.1 (0.33–2.64)	1.9 (0.92–5.18)	4.0 (2.02–7.36)	<0.0005

LDL, mean (SD)	3.5 (1.2)	3.3 (0.9)	3.2 (0.9)	3.2 (1.3)	0.3

Clinical heart failure, during hospitalization, %	18	16	30	62	<0.0005

PCI, %	31	32	21	12	0.009

CABG, %	19	11	12	11	0.4

LMWH or warfarin in hospital, %	94	88	89	80	0.04

Aspirin, at admission, %	41	55	45	43	0.3

Clopidogrel, at discharge, %	62	61	40	16	<0.005

Statins, at discharge, %	65	68	53	42	0.004

ACE inhibitors or ARBs, at discharge, %	36	49	49	61	0.02

Betablockers, at discharge, %	89	87	87	87	0.9

Thrombolysis	11	24	26	36	0.002

The aggregation of platelets increased during the hospital stay (p < 0.001) and was more pronounced with peak CRP levels in the fourth quartile (p < 0.0005) (Table 2). Closure time was also shorter, with higher levels of CRP (p < 0.05). The fourth quartile of peak CRP (>64.6 mg/l) was independently associated with increased platelet aggregation (p < 0.001, adjusted R^2 ^= 0.13), after adjustment for age, troponin T, platelet count, treatment with thrombolysis, the use of clopidogrel and LMWH. Furthermore, the fourth quartile of peak CRP was associated with an odds ratio of 2.2 (CI 95% 1.2–4.0) for high residual platelet reactivity (p = 0.003) in a univariate model and 2.9 (CI 95% 1.3–6.8) in a multivariate model, adjusted for age, platelet count, von Willebrand factor, treatment with thrombolysis, the use of clopidogrel and the use of LMWH. There was a small negative correlation between peak CRP and closure time, rho = -0.17, p = 0.002.

**Table 2:** Comparison of platelet aggregation, closure times (CT) and troponin T (TnT) levels according to quartiles of peak C-reactive protein (CRP)

	Quartile 1	Quartile 2	Quartile 3	Quartile 4	p
CRP, mg/l	0.05–5.3	5.4–19.6	19.7–64.5	64.6–304	

Peak SPA, count, ×10^3^	71 (22–91)	82 (30–120)	104 (45–150)	117 (70–160)	P < 0.0005

CT, sec	259 (150–300)	231 (143–300)	184 (137–300)	162 (124–300)	P < 0.05

TnT, μg/L	0.49 (0.21–1.26)	1.09 (0.33–2.64)	1.93 (0.92–5.18)	3.99 (2.02–7.36)	P < 0.0005

SPA = Small platelet aggregates. Peak SPA = The highest measured count of small platelet aggregates during hospitalization. Values are expressed as medians with the interquartile range in parentheses. Calculations using Kruskall-Wallis test.

During a median follow-up period of 44 months (interquartile range 35–55 months), 141 primary endpoint events occurred (ischemic cerebral event, myocardial infarction, or death of any cause). Median follow-up time according to quartiles of CRP was fifty-two, fifty, forty-four and thirty-nine months in the first, second, third and fourth quartile, respectively. No patients were lost to follow up. During the entire follow-up period a total of 28 cases of ischemic cerebral events, 65 myocardial infarctions and 88 deaths occurred. The rates for the combined endpoint of stroke, AMI and death in the different quartiles of peak CRP were twenty-four (28.2%), thirty-one (37.8%), thirty-four (40.5%) and fifty-two (62.7%) in the first, second, third and fourth quartile, respectively (p < 0.0005).

**Figure 1:**
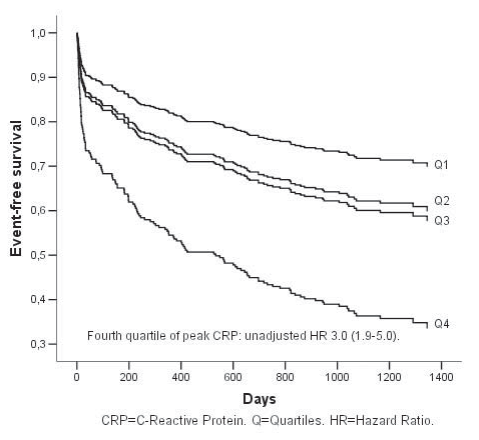
**Cumulative event-free survival (AMI, stroke and all death) in relation to peak CRP quartiles**. Univariate Cox-regression analysis.

**Figure 2:**
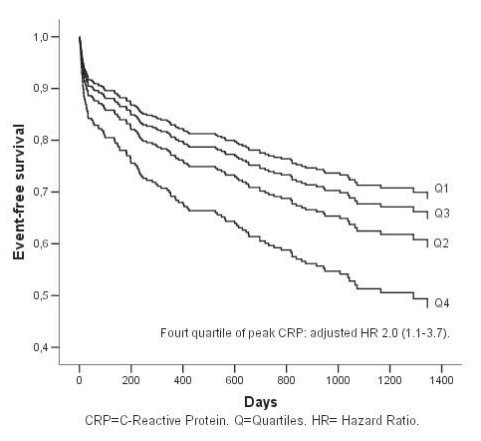
**Cumulative event-free survival (AMI, stroke and all death) in relation to peak CRP quartiles**. Multivariate Cox-regression analysis.

The hazard ratios (HR) for the prognostic variables are given in Table 3. The fourth quartile of peak CRP was associated with poorer outcome in a univariate model with a hazard ratio of 3.0 (95% CI 1.9–5.0) for the occurrence of ischemic cerebral event, myocardial infarction and death (Figure 1), as compared to the first quartile. Peak CRP predicted a worse outcome also after multivariate adjustment for age, sex, diabetes, smoking status, heart failure, atrial fibrillation, baseline glomerular filtration rate, troponin T, platelet aggregation, high residual platelet reactivity, intervention with CABG or PCI with a hazard ratio of 2.0 (95% CI 1.1–3.7) (Figure 2). Age, atrial fibrillation and clinical heart failure also predicted a worse prognosis (Table 3). Clinical heart failure had the second strongest association with poorer outcome with a hazard ratio of 1.8 (95% CI 1.2–2.6.

**Table 3:** Hazard ratios (Cox regression analysis) for the combined endpoint (acute myocardial infarction, stroke, death of all causes) for the entire follow-up period

Variable	Univariate	Multivariate
	Hazard ratio (95% Confidence interval)	

C-reactive protein		

Quartile 4 (Quartile 1 is reference)	3.0 (1.9–5.0)	2.0 (1.1–3.7)

Age (continues variable)	1.06 (1.0–1.1)	1.05 (1.0–1.1)

Sex		

Male	1.0	1.0

Female	1.3 (0.9–1.8)	0.9 (0.6–1.3)

Current smoker		

No	1.0	1.0

Yes	0.9 (0.6–1.4)	1.6 (1.0–2.6)

Diabetes		

No	1.0	1.0

Yes	1.6 (1.1–2.4)	1.5 (1.0–2.4)

Clinical heart failure During hospitalization		

No	1.0	1.0

Yes	3.1 (2.2–4.4)	1.8 (1.2–2.6)

Atrial fibrillation		

No	1.0	1.0

Yes	2.3(1.6–3.3)	1.6 (1.1–2.4)

Glomerular filtration rate		

>60 ml/min	1.0	1.0

<60 ml/min	2.5 (1.8–3.5)	1.2 (0.8–1.7)

Troponin T, quartile 4 (Quartile 1 is reference)	1.7 (1.1–2.7)	1.0 (0.6–1.8)

Platelet Aggregation		

Quartile 4 (Quartile 1 is reference)	2.0 (1.2–3.3)	1.1 (0.6–2.0)

High residual platelet reactivity		

No	1.0	1.0

Yes	0.8 (0.5–1.1)	0.7 (0.5–1.0)

CABG		

No	1.0	1.0

Yes	0.5 (0.3–0.9)	0.5 (0.3–1.0)

PCI		

No	1.0	1.0

Yes	0.4 (0.3–0.7)	1.0 (0.6–1.8)

**Figure 3:**
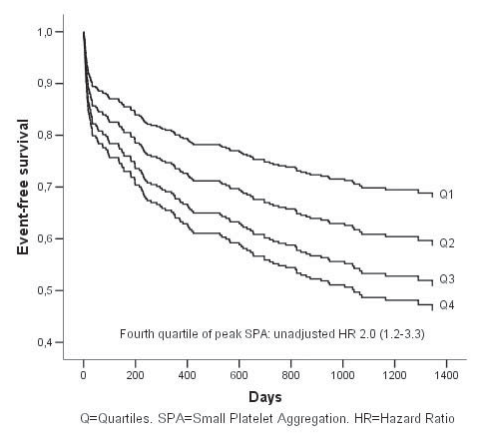
**Cumulative event-free survival (AMI, stroke and all death) in relation to peak small platelet aggregation quartiles**. Univariate Cox-regression analysis.

**Figure 4:**
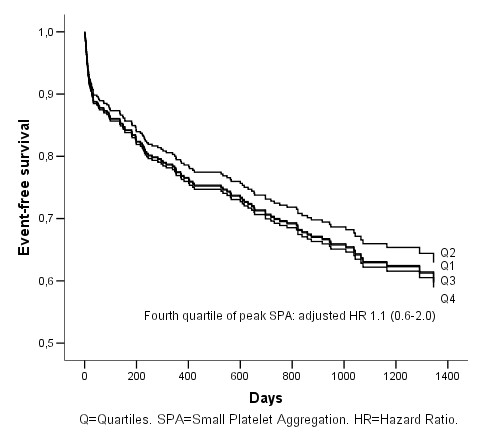
**Cumulative event-free survival (AMI, stroke and all death) in relation to peak small platelet aggregation quartiles**. Multivariate Cox-regression analysis.

The fourth quartile peak small platelet aggregates was associated with poorer outcome in a univariate Cox-regression analysis, HR 2.0 (CI 95% 1.2–3.3) (Figure 3), but when adjustment was made for the same co-variables as above the association was no longer there; HR 1.1 (CI 95% 0.6–2.0) (Figure 4). The state of high residual platelet reactivity did not predict poorer outcome in univariate or multivariate analysis.

## Discussion

This study shows that peak CRP is an independent predictor of prognosis in a population of patients with AMI. In a univariate analysis platelet aggregation was associated with worse prognosis but not in an adjusted analysis model. PFA-100 closure times give no prognostic information. Obviously, the potential prognostic value of platelet function tests has to be verified in prospective studies with careful adjustment for relevant clinical variables.

Peak CRP had a weak association with more pronounced peak platelet aggregation and lower PFA-100 closure times. The question whether CRP is a marker or involved in the mechanisms of atherothrombosis is still under debate, and more research is needed [28]. A better understanding of the mechanisms involved would open for further therapeutic improvements and more individualized treatment regimens.

Our results add to previous knowledge about CRP as a predictor of prognosis after an AMI. Suleiman et al. showed that CRP was an independent predictor of heart failure and mortality in 1044 survivors of acute myocardial infarction [29]. They measured CRP once and within 12 to 24 h of symptom onset, while in the present study peak CRP levels during the hospital stay were analyzed. We used peak CRP since it might better reflect the magnitude of the inflammatory response to myocardial necrosis, which we hypothesized would have an impact on platelet reactivity. Peak CRP was only significantly associated with death among the single endpoints which could be a power problem.

In contrast, the study by Bennermo et al. concluded that CRP after an ST-elevation myocardial infarction treated with thrombolysis does not carry any predictive value in a multivariate analysis. They did not, however, present any power calculation and their negative results might have been due to a small study population since only 45 primary events occurred [30]. Most of the previous studies in this area are sub-studies of randomized controlled trials and therefore contain a highly selected patient sample. Our study is an observational cohort study, not part of a clinical trial, which is reflected in the presence of more women and older patients.

We failed to show any substantial association between outcome and platelet aggregation or the state of high residual platelet reactivity. These findings do not support the idea that more pronounced platelet aggregation during hospitalization plays a long term key role in subsequent cardiovascular events. The fact that platelet aggregation for the whole study population peaked in average on the third day and was lower on day five might theoretically indicate that pronounced platelet aggregation have the strongest impact on adverse events in the early phase of a myocardial infarction and less importance later on. This theory is supported by the COMMIT trial, which showed that adding clopidogrel to aspirin only during a mean time of 15 days in patients with myocardial infarction reduced mortality [31].

Somewhat surprisingly, the state of high residual platelet reactivity was not associated with poorer prognosis, despite its relation to the systemic inflammatory response. This finding is in contrast to a meta-analysis of "aspirin resistance" by Krasopoulos et al., who concluded that "aspirin resistant" patients are at greater risk of adverse cardiovascular events [32]. Their metaanalysis however, was a composite of various platelet function tests and clinical index episodes. Furthermore, only two studies used the PFA-100 in acute coronary syndrome and neither of these had a prospective design [33,34]. Two studies used different aggregometry techniques in acute coronary syndrome, and one showed no relationship between platelet aggregation and cardiovascular events. The second study, which used cationic propyl gallate platelet aggregometry, showed an almost two-fold increased frequency of new cardiovascular events during a 4-year follow up. They did not, however, adjust for important clinical entities in their statistics, such as the occurrence of diabetes, smoking status, kidney function, heart failure, and so on. In addition, several other studies show a prognostic value of PFA-100, but most of the prospective studies included patients with chronic conditions, and not ACS patients with signs of systemic inflammatory response. Differences in patient populations and lack of adjustment for confounding variables are important reasons for the variance in the results [35]. In our study, we recorded 141 endpoints. The number of endpoints in previous studies was fairly low, ranging from three to just over one hundred, indicating a power problem.

The present study does not support an independent predictive value of PA-200 or PFA-100 results in terms of future cardiovascular events. On the contrary, our results reinforces the importance of clinical variables such as heart failure, age, atrial fibrillation, diabetes and smoking status all of which were associated with worse prognosis in a multivariate analysis.

## Limitations of the study

It is difficult to rule out a problem with insufficient power concerning the analysis of PA-200 and PFA-100 results as predictors of prognosis in the present study. Therefore, we might have failed to reject the null-hypothesis (type 2-error). The finding of positive results in previous studies with fewer participants might on the other hand have been due to chance (type 1-error), because it is generally believed that there is selection bias in relation to these types of studies, implying that papers with negative results are not published.

The association between CRP and cardiovascular events diminished substantially in the adjusted model. It is possible that there exist other factors of importance for prognosis that has not been adjusted for. We used clinical measures of heart failure as a prognostic variable. Although this variable was significantly related with prognosis a direct measurement of ventricular function might have added to the model.

Since this was not a controlled study we could not control, for example, for compliance to medication. This is an observational cohort study with its inherent limitations of possible confounding factors. The normal range of CT is wide and the appropriate cut off value for insufficiently inhibited platelets remains to be determined.

## Conclusion

Peak CRP during the course of an acute myocardial infarction is associated with new cardiovascular events during long-term follow up. Neither PFA-100 closure times nor peak platelet aggregation independently predict prognosis. There is an association between elevated CRP and more pronounced platelet aggregation as well as high residual platelet reactivity, the latter assessed using PFA-100. However, this study does not support the idea that the association between inflammation and subsequent cardiovascular events in the long term goes via more pronounced platelet aggregation or by high residual platelet reactivity. To adequately explore the relationships in the short term, a larger study is needed. Clinical variables retain important prognostic information.

## Competing interests

The authors declare that they have no competing interests.

## Authors' contributions

TM designed the study. All authors analyzed data and wrote the manuscript. All authors read and approved the final manuscript.

## References

[B1] Cohen M, Antman EM, Murphy SA, Radley D (2002). Mode and timing of treatment failure (recurrent ischemic events) after hospital admission for non-ST segment elevation acute coronary syndromes. Am Heart J.

[B2] Mooe T, Eriksson P, Stegmayr B (1997). Ischemic stroke after acute myocardial infarction. A population-based study. Stroke.

[B3] Mooe T, Olofsson BO, Stegmayr B, Eriksson P (1999). Ischemic stroke. Impact of a recent myocardial infarction. Stroke.

[B4] Witt BJ, Ballman KV, Brown RD, Meverden RA, Jacobsen SJ, Roger VL (2006). The incidence of stroke after myocardial infarction: a meta-analysis. Am J Med.

[B5] Tavazzi L (1999). Clinical epidemiology of acute myocardial infarction. Am Heart J.

[B6] Hellermann JP, Goraya TY, Jacobsen SJ, Weston SA, Reeder GS, Gersh BJ, Redfield MM, Rodeheffer RJ, Yawn BP, Roger VL (2003). Incidence of heart failure after myocardial infarction: is it changing over time?. Am J Epidemiol.

[B7] Jokhadar M, Jacobsen SJ, Reeder GS, Weston SA, Roger VL (2004). Sudden death and recurrent ischemic events after myocardial infarction in the community. Am J Epidemiol.

[B8] Guidry UC, Evans JC, Larson MG, Wilson PW, Murabito JM, Levy D (1999). Temporal trends in event rates after Q-wave myocardial infarction: the Framingham Heart Study. Circulation.

[B9] McGovern PG, Jacobs DR, Shahar E, Arnett DK, Folsom AR, Blackburn H, Luepker RV (2001). Trends in acute coronary heart disease mortality, morbidity, and medical care from 1985 through 1997: the Minnesota heart survey. Circulation.

[B10] Hedlund P (1961). Clinical and experimental studies on C-reactive protein (acute phase protein). Acta Med Scand Suppl.

[B11] Balbay Y, Tikiz H, Baptiste RJ, Ayaz S, Sasmaz H, Korkmaz S (2001). Circulating interleukin-1 beta, interleukin-6, tumor necrosis factor-alpha, and soluble ICAM-1 in patients with chronic stable angina and myocardial infarction. Angiology.

[B12] Brunetti ND, Troccoli R, Correale M, Pellegrino PL, Di Biase M (2006). C-reactive protein in patients with acute coronary syndrome: correlation with diagnosis, myocardial damage, ejection fraction and angiographic findings. Int J Cardiol.

[B13] Deng P, Zhao SP, Dai HY, Guan XS, Huang HG (2006). Atorvastatin reduces the expression of COX-2 mRNA in peripheral blood monocytes from patients with acute myocardial infarction and modulates the early inflammatory response. Clin Chem.

[B14] Danesh J, Collins R, Appleby P, Peto R (1998). Association of fibrinogen, C-reactive protein, albumin, or leukocyte count with coronary heart disease: meta-analyses of prospective studies. Jama.

[B15] Lagrand WK, Visser CA, Hermens WT, Niessen HW, Verheugt FW, Wolbink GJ, Hack CE (1999). C-reactive protein as a cardiovascular risk factor: more than an epiphenomenon?. Circulation.

[B16] Morrow DA, Rifai N, Antman EM, Weiner DL, McCabe CH, Cannon CP, Braunwald E (1998). C-reactive protein is a potent predictor of mortality independently of and in combination with troponin T in acute coronary syndromes: a TIMI 11A substudy. Thrombolysis in Myocardial Infarction. J Am Coll Cardiol.

[B17] Ridker PM, Cushman M, Stampfer MJ, Tracy RP, Hennekens CH (1997). Inflammation, aspirin, and the risk of cardiovascular disease in apparently healthy men. N Engl J Med.

[B18] (2000). Myocardial infarction redefined – a consensus document of The Joint European Society of Cardiology/American College of Cardiology Committee for the redefinition of myocardial infarction. Eur Heart J.

[B19] Kundu SK, Heilmann EJ, Sio R, Garcia C, Davidson RM, Ostgaard RA (1995). Description of an in vitro platelet function analyzer – PFA-100. Semin Thromb Hemost.

[B20] Franchini M (2005). The platelet function analyzer (PFA-100): an update on its clinical use. Clin Lab.

[B21] Cattaneo M (2007). Laboratory detection of 'aspirin resistance': what test should we use (if any)?. Eur Heart J.

[B22] Ozaki Y, Satoh K, Yatomi Y, Yamamoto T, Shirasawa Y, Kume S (1994). Detection of platelet aggregates with a particle counting method using light scattering. Anal Biochem.

[B23] Satoh K, Ozaki Y, Qi R, Yang L, Asazuma N, Yatomi Y, Kume S (1996). Factors that affect the size of platelet aggregates in epinephrine-induced activation: a study using the particle counting method based upon light scattering. Thromb Res.

[B24] Eto K, Takeshita S, Ochiai M, Ozaki Y, Sato T, Isshiki T (1998). Platelet aggregation in acute coronary syndromes: use of a new aggregometer with laser light scattering to assess platelet aggregability. Cardiovasc Res.

[B25] Sakamoto T, Ogawa H, Kawano H, Hirai N, Miyamoto S, Takazoe K, Soejima H, Kugiyama K, Yoshimura M, Yasue H (2000). Rapid change of platelet aggregability in acute hyperglycemia. Detection by a novel laser-light scattering method. Thromb Haemost.

[B26] Miyamoto S, Ogawa H, Soejima H, Takazoe K, Sakamoto T, Yoshimura M, Kugiyama K, Yasue H (2000). Formation of platelet aggregates after attacks of coronary spastic angina pectoris. Am J Cardiol.

[B27] Miyamoto S, Kawano H, Sakamoto T, Soejima H, Kajiwara I, Shimomura H, Kojima S, Hokamaki J, Sugiyama S, Hirai N (2003). Formation of platelet microaggregates correlates with adverse clinical outcome in patients with coronary artery disease. Thromb Haemost.

[B28] Ridker PM (2007). C-reactive protein and the prediction of cardiovascular events among those at intermediate risk: moving an inflammatory hypothesis toward consensus. J Am Coll Cardiol.

[B29] Suleiman M, Khatib R, Agmon Y, Mahamid R, Boulos M, Kapeliovich M, Levy Y, Beyar R, Markiewicz W, Hammerman H, Aronson D (2006). Early inflammation and risk of long-term development of heart failure and mortality in survivors of acute myocardial infarction predictive role of C-reactive protein. J Am Coll Cardiol.

[B30] Bennermo M, Held C, Hamsten A, Strandberg LE, Ericsson CG, Hansson LO, Tornvall P (2003). Prognostic value of plasma C-reactive protein and fibrinogen determinations in patients with acute myocardial infarction treated with thrombolysis. J Intern Med.

[B31] Chen ZM, Jiang LX, Chen YP, Xie JX, Pan HC, Peto R, Collins R, Liu LS (2005). Addition of clopidogrel to aspirin in 45,852 patients with acute myocardial infarction: randomised placebo-controlled trial. Lancet.

[B32] Krasopoulos G, Brister SJ, Beattie WS, Buchanan MR (2008). Aspirin "resistance" and risk of cardiovascular morbidity: systematic review and meta-analysis. Bmj.

[B33] Borna C, Lazarowski E, van Heusden C, Ohlin H, Erlinge D (2005). Resistance to aspirin is increased by ST-elevation myocardial infarction and correlates with adenosine diphosphate levels. Thromb J.

[B34] Hobikoglu GF, Norgaz T, Aksu H, Ozer O, Erturk M, Nurkalem Z, Narin A (2005). High frequency of aspirin resistance in patients with acute coronary syndrome. Tohoku J Exp Med.

[B35] Gurbel PA, Becker RC, Mann KG, Steinhubl SR, Michelson AD (2007). Platelet function monitoring in patients with coronary artery disease. J Am Coll Cardiol.

